# Potential Applications of Endo-β-*N*-Acetylglucosaminidases From *Bifidobacterium longum* Subspecies *infantis* in Designing Value-Added, Next-Generation Infant Formulas

**DOI:** 10.3389/fnut.2021.646275

**Published:** 2021-04-09

**Authors:** Hatice Duman, Merve Kaplan, Ayşenur Arslan, Arif Sercan Sahutoglu, Haci Mehmet Kayili, Steven A. Frese, Sercan Karav

**Affiliations:** ^1^Department of Molecular Biology and Genetics, Çanakkale Onsekiz Mart University, Çanakkale, Turkey; ^2^Department of Chemistry, Çanakkale Onsekiz Mart University, Çanakkale, Turkey; ^3^Department of Biomedical Engineering, Karabuk University, Karabük, Turkey; ^4^Department of Nutrition, University of Nevada, Reno, NV, United States; ^5^Department of Food Science and Technology, University of Nebraska Lincoln, Lincoln, NE, United States

**Keywords:** human milk oligosaccharides, *N*-glycans, endo-β-*N*-acetylglucosaminidase, bifidobacteria, infant formula

## Abstract

Human milk is the optimal source of infant nutrition. Among many other health benefits, human milk can stimulate the development of a *Bifidobacterium*-rich microbiome through human milk oligosaccharides (HMOs). In recent years, the development of novel formulas has placed particular focus on incorporating some of the beneficial functional properties of human milk. These include adding specific glycans aimed to selectively stimulate the growth of *Bifidobacterium*. However, the bifidogenicity of human milk remains unparalleled. Dietary *N-*glycans are carbohydrate structures conjugated to a wide variety of glycoproteins. These glycans have a remarkable structural similarity to HMOs and, when released, show a strong bifidogenic effect. This review discusses the biocatalytic potential of the endo-β-*N*-acetylglucosaminidase enzyme (EndoBI-1) from *Bifidobacterium longum* subspecies *infantis (B. infantis)*, in releasing *N-*glycans inherently present in infant formula as means to increase the bifidogenicity of infant formula. Finally, the potential implications for protein deglycosylation with EndoBI-1 in the development of value added, next-generation formulas are discussed from a technical perspective.

## Introduction

Human milk is the optimal source of infant nutrition. It provides all the energy, nutrients, and bioactive compounds required for the growth and development of the infant. Human milk feeding is associated with numerous benefits, including a reduced risk of gastrointestinal and respiratory infections and improved immune development (1). Given the known benefits of human milk, there is a great interest in improving infant formulas to resemble the compositional profile of human milk (2) and reduce the relative deficits associated with infant formula consumption. Thus, a better understanding of human milk components and their biological functions is paramount to the improvement of infant formulas (3, 4).

One of the most significant differences between human milk-fed and formula-fed infants is the composition of the gut microbiome (4–6). Breastfed infants have a less diverse yet more stable microbiome, and certain species of infant-adapted bifidobacteria can reach up to 90% of total fecal microbiome (7–9). On the other hand, the microbiome of the formula-fed infants is more variable (8, 10). To mitigate these differences between infant formula and human milk, most formulations add prebiotics such as galacto-oligosaccharides (GOS) and fructo-oligosaccharides (FOS) (11, 12) and/or probiotics. Probiotics added to formula are currently limited to *Lactobacillus rhamnosus* GG and *Bifidobacterium lactis* (13). Human milk contains complex carbohydrates known as human milk oligosaccharides (HMOs). HMOs are not digested in the small intestine and reach the colon intact where they are fermented by specialized species of bifidobacteria (14). However, most prebiotic compounds added to formula are not selective for the growth of bifidobacterial (15). Thus, the difference in oligosaccharide content in human milk and infant formula is likely to explain, at least in part, the compositional differences in the microbiome of formula-fed and human milk-fed infants.

Recently, synthetic HMOs such as 2′-fucosyllactose (2′FL) and lacto-*N-*neotetraose (LNnT) have been added to infant formula with the intent to increase the bifidogenic effect of infant formula (16–19). However, HMO fortification of infant formulas has remained low when compared to the global average concentration of HMOs in human milk. On the other hand, little attention has thus far been given to *N-*glycans, which are naturally found as glycoconjugates in both human and bovine milk proteins and bear striking structural and compositional similarity to HMOs. Owing to both their compositional and structural similarities to HMOs, *N-*glycans derived from milk glycoproteins have been shown to be selectively bifidogenic. In this review, we describe human milk as a complex biofluid. We then describe the types, compositions, and indications for most infant formulas available in the market. Finally, we propose the use of specialized enzymes known to be active in the gut microbiome of breastfed infants colonized with *Bifidobacterium* in order to improve the bioavailability of *N-*glycans in infant formula and we discuss potential applications for the design on next-generation infant formulas to improve the suitability of infant formulas for *Bifidobacterium*.

## Macronutrients in Human Milk

The composition of human milk is dynamic, and it has evolved to provide optimal infant nutrition. Human milk contains macronutrients including proteins, lipids, carbohydrates, and micronutrients such as vitamins and minerals. It also contains non*-*nutritional bioactive components, growth factors, hormones, immunological factors, noncoding RNAs, and microorganisms (20). The macronutrient composition of human milk ranges from 9 to 12 g/L protein, 32 to 36 g/L lipids, 67 to 78 g/L lactose, and 5 to 15 g/L HMOs (3, 21, 22) (Table 1).

**Table 1:** Human milk composition.

**Component**	**Amount**	**References**
**Human milk composition**		
**Energy**	65–70 kcal/dL	(3)
**Lactose**	67–78 g/L	(3)
**Protein**	9–12 g/L	(3)
**Lipid**	32–36 g/L	(3)
**Vitamins**		
Vitamin D	4–40 IU/L	(23)
Vitamin C	30.3 ± 6.7 mg/L	(24)
Vitamin K	0.9–6.9 mg/L	(23)
**Minerals**		
Calcium	84–462 mg/L	(25)
Magnesium	15–64 mg/L	(26)
Phosphorus	17–278 mg/L	(25)
Sodium	512 mg/L	(23)
**HMOs**	5–15 g/L	(21, 22, 27)
Lactose		
2′-Fucosyllactose (2′FL)		
3′-Fucosyllactose (3′SL)		
6′-Siayllactose (6′SL)		
3′-Sialyllactose (3′SL)		
Lacto-*N-*tetraose (LNT)		
Lacto-*N*-neotetraose (LNnT)		
Lacto-N-hexaose (LNH)		
Lacto-*N*-fucopentaose I (LNFP I)		
Lacto-*N*-fucopentaose II (LNFP II)		
Lacto-*N*-fucopentaose III (LNFP III)		
Lacto-*N*-fucopentaose V (LNFP V)		
Siayllactose-*N*-tetraose b (LST b)		
Siayllactose-*N*-tetraose c (LST c)		
Disiayllacto-*N*-tetraose (DSLNT)		
Fucosyllacto-*N*-hexaose (FLNH)		
Difucosyllacto-*N*-hexaose (DFS-LNH)		

Proteins in human milk comprise two major classes, caseins, and whey (28). The main casein proteins are α-, β-, and κ-casein, and whey proteins are α-lactalbumin, lactoferrin, immunoglobulins (Igs), serum albumin, and lysozyme (29, 30). Non*-*protein nitrogen*-*containing compounds including urea, uric acid, creatine, creatinine, amino acids, and nucleotides represent ~25% of human milk nitrogen (31).

Fat is the largest source of energy in human milk, contributing to 40–55% of the total energy provided by human milk. Triacylglycerols contribute ~98% of human milk fat. More than 200 fatty acids are present in human milk with different concentrations (32). Palmitic and oleic acids are the most abundant fat types in human milk (33). The content of fatty acids, particularly the long-chain polyunsaturated fatty acids (LCPUFAs), is mostly affected by maternal diet.

Lactose is the main nutritional carbohydrate in human milk comprising 67–78 g/L and supplies approximately half of the energy obtained in by the infant. The other significant carbohydrate fractions of human milk are HMOs. However, contrary to that of lactose, the concentration of HMOs varies depending on the stage of lactation and maternal genetic factors, ranging from 5 to 15 g/L (34).

## Human Milk Oligosaccharides (HMOs)

HMOs are non*-*nutritive, functional, and complex carbohydrates in human milk. The composition of HMOs in human milk is influenced by maternal genetic and lactation stage (35). Nearly 200 distinct oligosaccharides have been described to date (36). The basic core structure of HMOs includes disaccharide lactose at the reducing end, which is elongated with *N-*acetyllactosamine units, by the action of specific glycosyltransferases in the mammary gland. HMOs are composed of both neutral and anionic species with five monosaccharides as building blocks. These building blocks are D-glucose (Glc), D-galactose (Gal), *N-*acetylglucosamine (GlcNAc), L-fucose (Fuc), and *N-*acetylneuraminic (or sialic acid; NeuAc). The length of the HMO chains varies from three to fifteen carbohydrate units, and HMO structures can be either linear or branched forms (37, 38). There are three main HMO categories: neutral *N-*containing (non*-*fucosylated) (42–55%), neutral (fucosylated) (35–50%), and acidic (sialylated) (12–14%) (39).

2′-3-Fucosyllactose (FL) or 3′-6′-sialyllactose (SL) is formed when the lactose core is conjugated with Fuc or NeuAc. The lactose core is coupled to repeats of lacto-*N-*biose (Galβ1-3GlcNAc; LNB), and these chains are known as type 1 chains. The most abundant HMO is lacto-*N-*tetraose (LNT) as type 1 (40). When an *N-*acetyllactosamine unit (LacNAc; Galβ1-4GlcNAc) is conjugated to the lactose core, the type 2 chain is formed. Lacto-*N-*neotetraose (LNnT) is a type 2 chain in HMOs. Type 1 chains in HMOs are more abundant than those of type 2. Type 1 and 2 chain HMOs could be further elongated with fucosyl and sialyl residues in α-linkages to form hexoses, octaoses, and larger HMOs and together represent ~70% of all human milk oligosaccharides (34, 41) (Table 1). These alterations increase the number and complexity of HMO structures (38, 42).

### Functions of HMOs

HMOs are hypothesized to have many important roles in infant innate defense, metabolic health, and neural development (43–45). Clinical and *in vitro* studies suggest that HMOs may block pathogen adhesion by serving soluble ligand analogs (43, 46, 47). As HMOs have structural features that mimic epithelial surface carbohydrates, they are thought to also serve as decoy receptors for pathogens (46, 48–50). HMOs are also thought to promote several intracellular processes like differentiation and apoptosis of intestinal epithelial cells (51). They can also have direct bactericidal or bacteriostatic effects. For instance, some HMOs can directly inhibit the *in vitro* growth of *Streptococcus agalactiae*, a known invasive bacterial pathogen in newborns (27, 52); other HMOs have been demonstrated to reduce pathogen adherence to colonic cells *in vitro* (53). Specific components present in HMOs (e.g., sialic acid) are also critical for the development of neurons and brain development, as well as neuronal transmission, cognitive ability and synaptogenesis (45, 54, 55).

One of the most well-characterized functions of HMOs is to serve as a prebiotic source and shape the microbial community of the infant gastrointestinal tract (56). HMOs reach the colon undigested where they are utilized by specialized gut microbes (57) that possess the necessary molecular machinery for transport and metabolization of these complex structures. Specific species of infant-adapted bifidobacteria [*Bifidobacterium longum* subsp. *infantis* (*B. infantis*), *Bifidobacterium bifidum* (*B. bifidum*), *Bifidobacterium breve* (*B. breve*), and *Bifidobacterium longum* subsp. *longum* (*B. longum*)] have the capability to degrade and utilize oligosaccharides and thus often become the most dominant species in the breastfed infant gut (58–61). Short-chain fatty acids (SCFA) (acetate, propionate, and butyrate) are produced as a result of fermentation of HMO in the colon. These molecules create an acidic environment (low pH) which favors the growth of strains of bifidobacteria while concomitantly creating an unfavorable environment for the growth of pH-sensitive pathogens (7, 41, 62).

## Human Milk Glycoproteins and their Functions

Glycosylation is a diverse and common type of posttranslational modification that involves the attachment of a saccharide chain to a protein structure (63, 64). Approximately 70% of human milk proteins are found in glycosylated forms including lactoferrin, lysozyme, bile salt-stimulated lipase (BSSL), secretory IgA (SIgA), casein, and α-lactalbumin (65, 66). Several preclinical and clinical studies suggest that human milk glycoproteins have key roles in infant development. For instance, osteopontin is involved in regulating mineral deposition and osteoclasts activity in the bones (67); insulin*-*like growth factors participate in the processes related to the development of the intestinal mucosa (68); bile salt-stimulated lipase aids milk fat digestion (69); lactoferrin facilitates iron uptake in the small intestine (70); and β-casein-based phosphopeptides facilitate calcium absorption (71, 72).

Human milk glycoproteins may also have roles in protecting infants against pathogen infection (73–75). Lactoferrin has been reported to have bacteriostatic and bactericidal effects (76, 77). Lysozyme cleaves glycosidic linkage in the peptidoglycan structure of bacterial cell walls, providing innate protection against microbial infections (78). Interestingly, the level of lysozyme susceptibility varies between different bifidobacteria strains (79, 80). Some bifidobacteria strains of human infant origin are more resistant to lysozyme relative to animal and dairy-derived strains (81). This may suggest that lysozyme in human milk acts as a selection factor for coevolved bifidobacteria in the infant gut, such as *B. infantis* (80, 82, 83). Another predominant human milk protein is SIgA. SIgA acts as a protective defense against pathogens in the infant gut (74, 84). Other human milk glycoproteins, including BSSL and lactadherin, also have protective effects on the infant's health (74). Notably, BSSL has been associated with inhibition of Norwalk virus, a common cause of gastroenteritis, *in vitro* (85).

**Figure 1:**
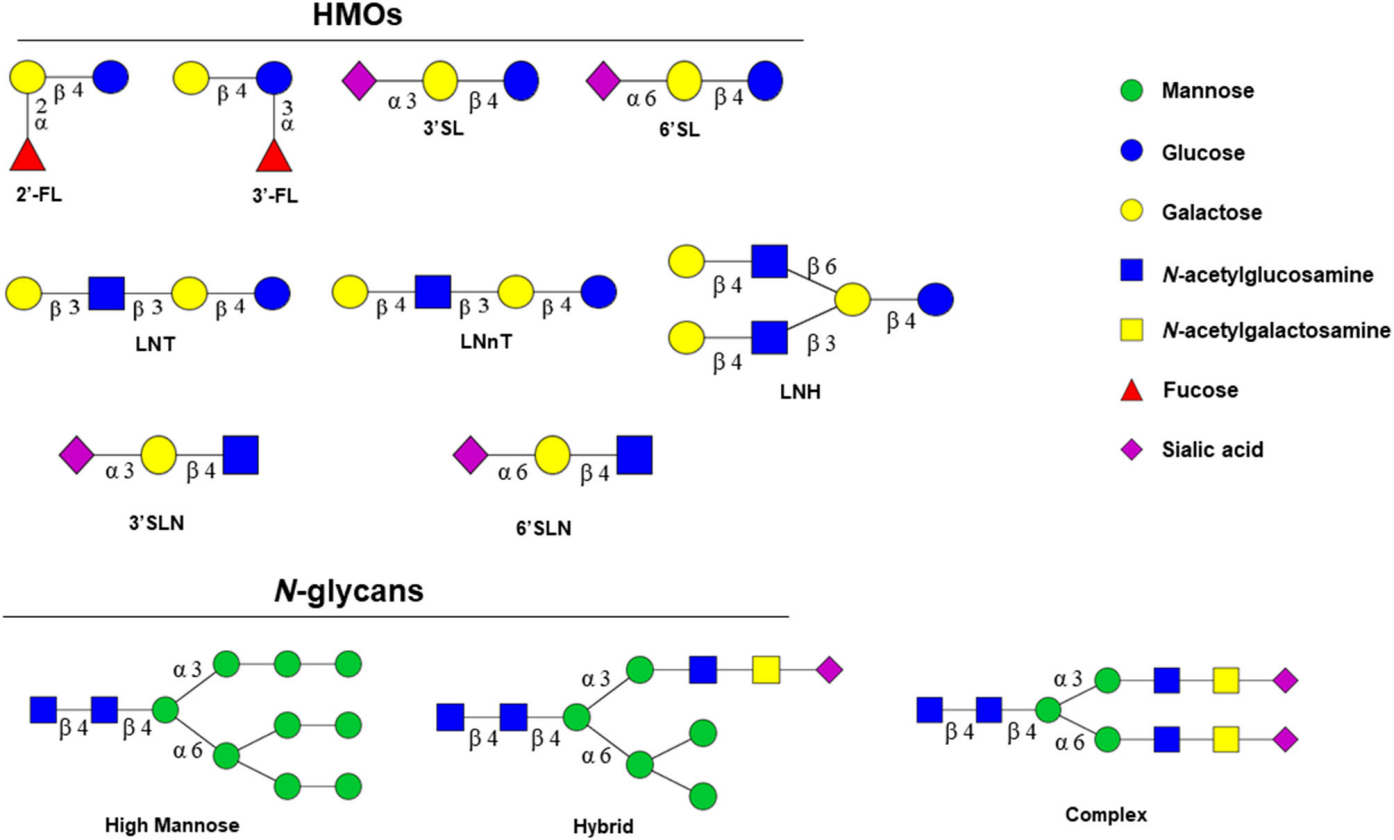
Structural similarities between HMOs and *N-*glycans. Structurally, *N*-glycans are bound to the amide group of asparagine (Asn) residue of the proteins via *N-*acetylglucosamine (HexNAc) in a specific amino acid sequence Asn*-*X-Ser/Thr or Asn*-*X-Cys (cysteine) (where X could be any amino acid except proline) (87, 88). *N*-glycans consist of a single core that has two *N-*acetylglucosamine (GlcNAc) followed by three mannoses. Further glycosylation determines the types of *N*-glycans that are classified into three main classes: high mannose (HM), hybrid (HY), and complex type (CT) based on composition (89); HM glycans typically contain unsubstituted terminal mannose sugars and 5–9 mannose residues attached to the chitobiose (GlcNAc_2_) core.

The glycan structures found on these glycoproteins are strikingly similar to HMOs, in both their monosaccharide composition and linkage types (86) (Figure 1). *N-*glycans also form complex structures which increase their specificity. This may explain why *N-*glycans isolated from human and bovine milk are bifidogenic (90), although not equally across bifidobacterial species (91). Specifically, *N-*glycans released from bovine milk glycoproteins selectively stimulates the growth of infant-adapted *B. infantis* whereas *B. animalis*, associated with an animal origin, is not capable of utilizing these structures (91). Further, a recent *in vivo* study showed that 19 unique *N-*glycan structures that are attached to lactoferrin and immunoglobulins stimulate the growth of *B. infantis* (92). Similar to HMOs, *N*-glycans are fermented into SCFAs, mainly lactate, acetate, and also butyrate and propionate (93). The colonic epithelium and microbial ecosystem can be affected from these end products by absorbing SCFAs and lowering the pH of the ecosystem (93). These metabolites primarily lactate and acetate lower the intestinal pH providing resistance to microbial colonization (7, 62, 94). Importantly, fermentation of *N*-glycans into acidic end-products, such as acetate and lactate, disfavors the growth of bacteria that degrade gastrointestinal mucin, and contributes to a considerable reduction in potentially pathogenic bacteria (7, 94–96). This is because most pathogenic bacteria preferentially grow near neutral pH (pH: 6.0–7.0) or grow under acidic conditions inefficiently (97). Therefore, the establishment of the gut microbiome by limiting pathogenic bacterial composition maximizes nutrition for other microbes and reduces inflammation, virulence factors, and antibiotic-resistant genomes (ARGs) in the gut environment. Thanks to the results of the fermentation and these metabolites, colonization of probiotic bacterial level in the gut microbiome, especially *Bifidobacterium* and genes conferring utilization of *N*-glycans, significantly increases. Thus, the development of the gut microbiome by providing colonization resistance to intestinal pathogens is critical for the development of the infant gut microbiome (94, 98).

## Infant Formula and *N-*Glycans

Infant formulas are intended as an effective breast milk substitute and are formulated to mimic nutritional composition, including macro- and micronutrients as well as bioactive components, of human milk (99). Most infant formulas are manufactured from bovine milk. The nutritional composition of all infant formulas must follow the global standards as recommended by the European Society for Pediatric Gastroenterology, Hepatology, and Nutrition's (ESPGHAN) international expert group that was commissioned by The Codex Alimentarius Commission in November 2004 (100, 101).

There are several types of infant formulas (102, 103). Some have specific clinical indications for use, including special formulas for preterm infants, protein hydrolysate or elemental formulas for infants that have cow's milk and soy protein allergies, or formulas for other specific nutritional requirements. Other types of formula include indications such as lactose-free formulas for lactose-intolerant infants, soy formulas for galactosemia, and sensitive formulas that contain partially hydrolyzed or reduced lactose content (Table 2).

**Table 2:** Types of infant formulas and their properties.

**Type of formula**	**Key product features**	**Intended**
Routine use	Conventional protein, fat, and carbohydrate composition to support healthy growth and development, meeting the requirements, for example, of the US Infant Formula Act, 1980 (104)	Suitable for most term infants, when breast milk is not an option
Premature	May contain partially hydrolyzed whey and carbohydrate source lactose.	Premature and low birth weight infants, where donor milk or mother's own milk is not available
	Higher calcium, phosphorus	
Allergy management	1. Extensively hydrolyzed casein and/or whey	Infants with allergy based on cow milk protein, where breast milk is not an option
	2.100% free amino acids. No peptides	Infants with bovine milk protein hypersensitivity even with extensively hydrolyzed cow milk protein, and where breast milk is not an option
Specialized metabolic conditions where breast milk may not be an option	Carbohydrate-free formula	Infants with carbohydrate metabolism disorders and carbohydrate malabsorption
	Reduced and modified fat formula	Infants with fat malabsorption, chylothorax, and decreased bile salts
	Reduced mineral formula: lower phosphorus, iron, and potassium	Infants with calcium disorder, renal insufficiency

The development of infant formulas has advanced significantly over the past 50 years. Nonetheless, an “ideal” microbiome where *Bifidobacterium* species predominate cannot yet be obtained with infant formula feeding. Previously, we reported that *N-*glycans, which are released from cow's milk proteins, have prebiotic activity supporting the growth of *B. infantis* (90, 91). Thus, releasing *N-*glycans from proteins being added to infant formulas may be an innovative and effective strategy to harness the activity of naturally active enzymes in the microbiome of breastfed infants to enhance the bifidogenicity of infant formulas.

## Release of *N-*Glycans from Glycoproteins

*N-*glycans can be released by chemical and enzymatic methods (105). However, enzymatic release is considered a preferred method as it eliminates the possibility of chemical or residual contamination. Moreover, due to the highly specific nature of the enzymes, the enzymatic release of *N-*glycans represents a more targeted and efficient approach for releasing and increasing the bioavailability of these bifidogenic structures. There are two known enzymes that can release *N-*glycans: *N*-acetylglucosaminidases and endo-β*-N-*acetylglucosaminidases (ENGases).

ENGases belong to EC number 3.2.1.X which corresponds to the glycosylase-type hydrolyses cleaving *O-* and *S-*glycosyl compounds. ENGases are further classified according to their glycoside hydrolase (GH) family membership. These enzymes are classified into two groups, GH families 18 and 85, based on their amino acid sequence (106) within the Carbohydrate-Active enZymes (CAZy) Database (http://www.cazy.org) (107). Family GH18 is unusual in having glycoside hydrolases that are both catalytically active chitinases and ENGases and also subfamilies of non-hydrolytic proteins that function as carbohydrate-binding modules/ “lectins” or as xylanase inhibitors whereas family GH85 solely contains ENGases.

Although all of the ENGases carry out the same hydrolytic reaction, they have different tolerances as to the precise structure of the *N*-glycans that they can hydrolyze. The ENGases are all retaining glycosidases that hydrolyze substrates via a two-step mechanism involving general acid/base catalysis. The main difference between GH18 and GH85 ENGases is the active-site amino acids either being two carboxylic acid residues (Glu and Asp) or one carboxylic acid and one amino group (Glu and Asn), respectively. Regardless of whether the active site contains one or two carboxylic acids, the hydrolytic mechanism catalyzed by the ENGases involves neighboring group participation of the 2-acetamide of the second GlcNAc residue (108).

**Figure 2:**
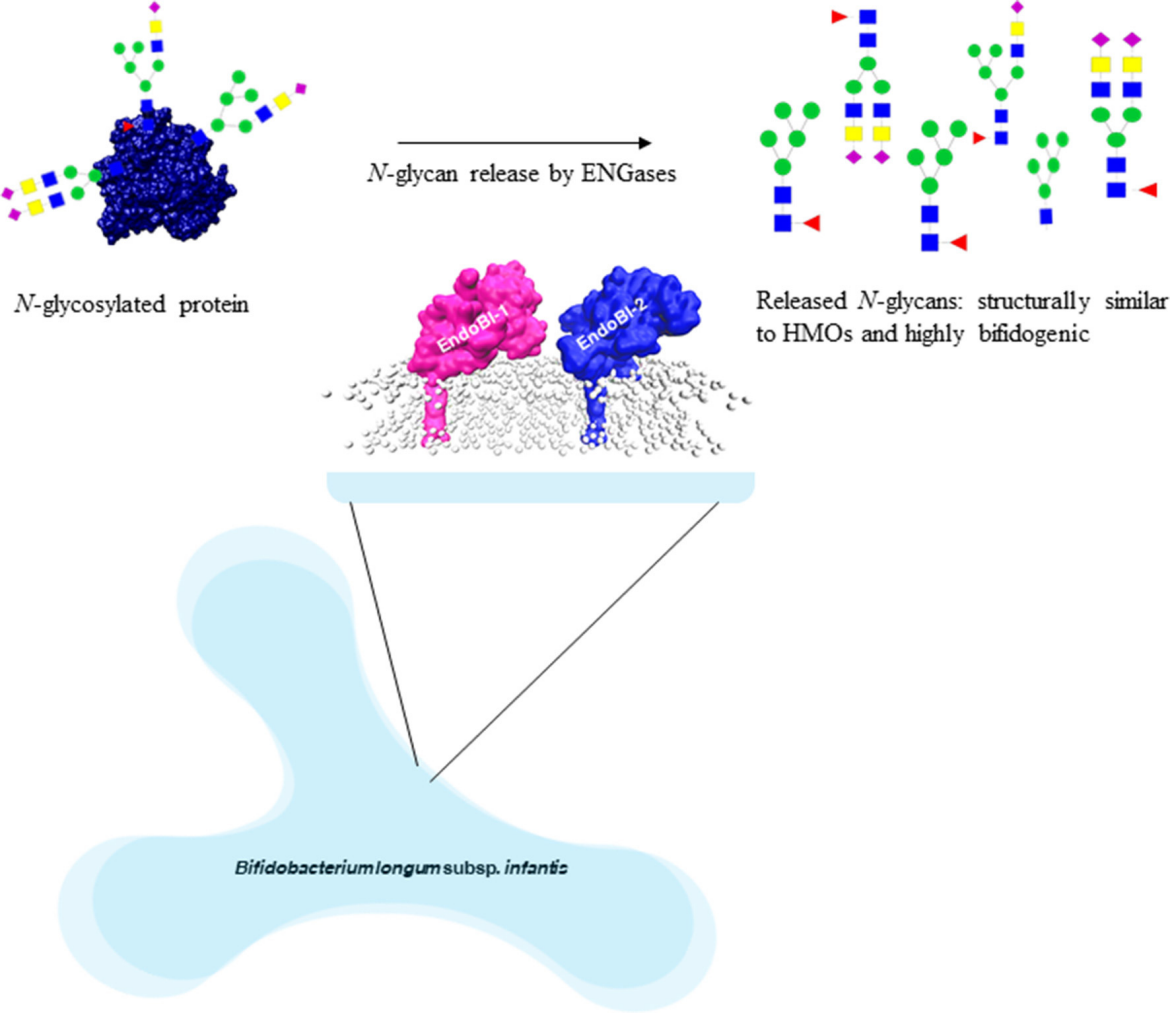
Formation of highly bifidogenic *N-*glycans by *B. infantis* ENGases from glycoproteins (91, 92).

ENGase enzymes cleave *N*-*N*′-diacetyl chitobiose moieties found in the *N*-glycan core of high mannose (HM), complex (CT), and hybrid (HY) *N*-glycans (Figure 1) and the released *N*-glycans that stimulate the growth of *B. infantis* (109) (Figure 2). EndoBI-1 from *B. infantis* (ATCC 15697) is a product of the Blon_2468 gene. Other *B. infantis* strains known to produce EndoBI-1 are JCM 7007, JCM 7009, JCM 7011, JCM 11346, ATCC 15702, and ATCC 17930 (110). The enzyme is classified as a GH20 member in the National Center for Biotechnology Information Genetic Sequence Database (NCBI-GenBank: ACJ53522.1) and EMBL European Bioinformatics Institute (EBI) European Nucleotide Archive (ENA CP001095.1) (111) and as a GH18 member in The Universal Protein Resource Knowledgebase (UniProtKB: B7GPC7) (110). The other ENGase, EndoBI-2 from *Bifidobacterium longum* subsp. *longum* 157F (deposited as *B. longum* subsp. *infantis* 157F), is a product of the BLIF_1310 gene (112, 113). The enzyme is classified as a GH18 member in NCBI-GenBank (BAJ71450.1). To date, only EndoBI-1 has been shown to be active in the gut of healthy breastfed infants colonized by *B. infantis* EVC001 (92), but both are likely to be expressed *in vivo*. Interestingly, EndoBI-1 and EndoBI-2 have different distributions among strains of *Bifidobacterium* found in infants compared to adults, which may further suggest the importance of these enzymes in healthy gut microbiome formation in both adults and infants (114).

EndoBI-1 and EndoBI-2 are unique among other ENGase members. EndoBI-1 and EndoBI-2 cleave *N*-glycans without perturbing the native glycan structure (115). The enzymes are considered fucose tolerant (110), meaning their activity is not affected by a fucosylated *N*-glycan core and therefore has a wider substrate specificity than similar enzymes (116). Both enzymes are active toward all major types of *N-*glycans found in glycosylated proteins (110). These unique enzymes are heat resistant, which enables broad applications even for industrial operations up to 95°C (110, 117), in contrast to the currently commercially available *N*-acetylglucosaminidases such as PNGase F of *Flavobacterium meningosepticum* which is heat labile (116). Further, both enzymes are considered safe for use in the food and pharmaceutical industries, especially when considering the sources of similar ENGase enzymes which are used by potential pathogens to evade the host immune system; such as Endo-COM from *Cordyceps militaris* (118), EndoS and EndoS2 from *Streptococcus pyogenes* (119, 120), EndoF3 from *Elizabethkingia meningoseptica* (121, 122), EndoH from *Streptomyces plicatus* (123, 124), EndoD from *Streptococcus pneumoniae* (3GDB.pdb), and EndoT from *Hypocrea jecorina* (125). Thus, making EndoBI-1 and EndoBI-2 the only two enzymes currently considered safe for food applications. Importantly, EndoBI-1 and EndoBI-2 could be easily cloned and/or mass produced with known microbiologic procedures and industrial techniques (110).

## Challenges in the Study and Characterization of *N*-Glycans

One of the primary challenges facing the translation of technologies surrounding *N*-glycan release is the precise and accurate quantification and characterization of *N*-glycans. Structural analyses of oligosaccharides and glycoconjugates by high-throughput approaches are crucial for predicting their functions. A number of chromatographic techniques have been employed for the analysis of oligosaccharides (126). One of the most common is porous graphitized carbon chromatography–mass spectrometry (PGC-MS) (127). This method can distinguish the isomers of oligosaccharides and *N-* and *O-*glycans of glycoconjugates with different linkage positions. This ability of PGC-MS makes the method more powerful than previous techniques. To achieve the structural identifications of HMOs faster and with more precision, a library was recently presented for both native and sialylated oligosaccharides, including retention times, accurate masses, and tandem mass spectra of HMOs (38, 42). In addition, relative and absolute quantification of HMOs was performed using the PGC-MS approach (128). Thus, the alterations of HMO profiles could be monitored throughout certain periods such as lactation. For example, a specific method was recently demonstrated for the absolute quantification of neutral and acidic HMOs (129). PGC-MS can also be used for the characterization of glycoconjugates of human milk. In one study, *N-*glycans released from human milk whey glycoproteins were analyzed and compared with bovine milk *N-*glycans using the PGC-MS technique (130). On the other hand, a method including solid-phase permethylation step was presented for the analysis of HMOs and glycans derived from human and bovine milk whey glycoproteins by reverse-phase liquid chromatography mass spectrometry (RPLC-MS) (131). Matrix-assisted laser desorption/ionization time-of-flight mass spectrometry (MALDI-MS) has also been commonly used for the characterization of HMOs and glycoconjugates (132, 133). This approach makes the analysis very fast when compared with chromatographic and electrophoretic techniques. In this approach, typically neutral HMOs and *N-/O-*glycans can be quantified using MALDI-MS because of unstable sialic acid residues found in HMOs and glycoconjugates. However, sialic acids can be derivatized by certain methods to make them more stable during the MALDI-MS analysis (134).

## Potential Application of ENGases in Next-Generation Formulas and Challenges

Although the composition of human milk is unparalleled in terms of suitability for infant nutrition, there are a number of logistical, practical, and medical reasons that necessitate the use for infant formulas with barriers to breastfeeding and racial inequities and/or socioeconomic barriers being prominent (135–138). The use of microbial enzymes is a staple of the industrial progress in the 21st century (139). The development of infant formula has not been the exception. For example, various next-generation infant formulas have been developed to manage cow milk protein allergy. Infant formulas with reduced allergenicity generally have partially or extensively hydrolyzed proteins, or amino acid-based formulations. Allergenicity is decreased by converting proteins to smaller peptides for modifying conformation or structure epitopes recognized by the immune system while maintaining caloric and protein and content, or by replacing intact proteins or peptides with amino acid formulations (140).

The process of producing partially or extensively hydrolyzed proteins involves complex proteolytic processing steps to reduce the size of bovine milk proteins. Protein glycosylation provides a stabilizing effect to proteins, making the native protein state more resistant to degradation (141, 142). Glycosylated proteins are more resistant to proteases compared to their aglycosylated (never glycosylated) or deglycosylated (enzymatically removed) counterparts (143–145). In fact, the rate of proteolysis and the amount of intact peptide (epitopes) available to reach up the intestinal tract are influenced by the presence of structural glycans (146). As bovine milk protein processing represents a major hurdle for the production of partially and extensively hydrolyzed proteins in infant formulas, the introduction of ENGases to this process has the potential to increase the efficiency and extent of protein hydrolysis required for infant formulas.

**Figure 3:**
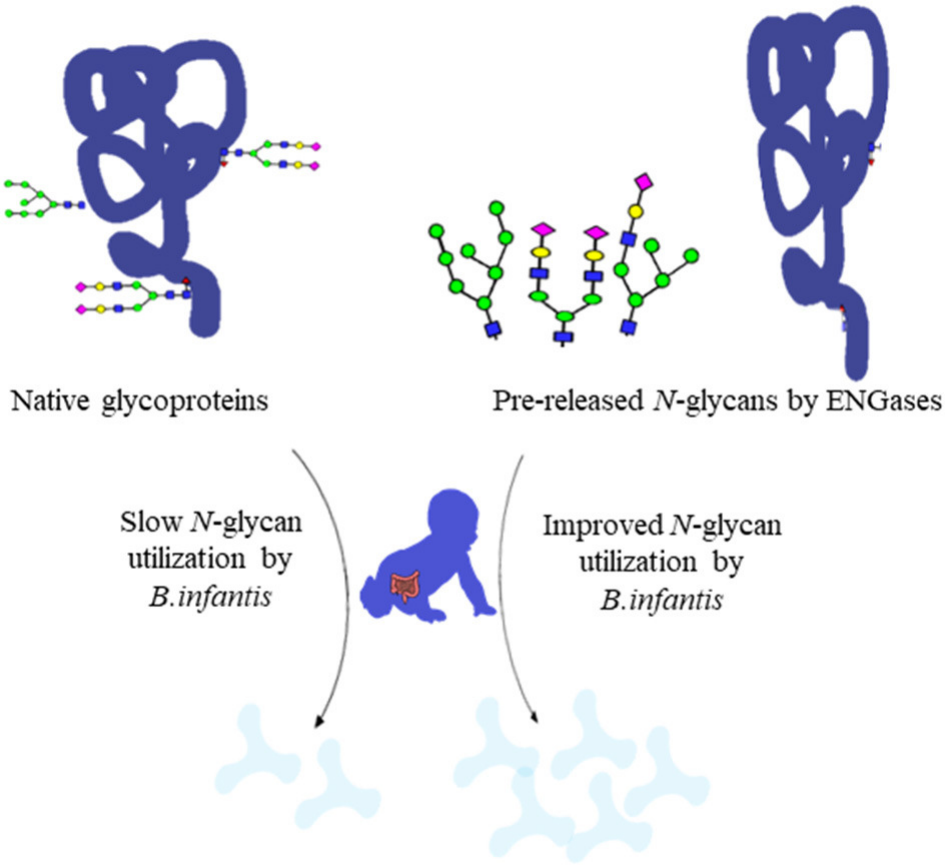
Utilization rate of native glycoproteins and pre-released glycans by ENGases (91).

Deglycosylation may also have implication for bioactive proteins and the released *N*-glycans. For instance, lactoferrin, an important bioactive protein added to formula, is heavily glycosylated. Modifying glycosylation patterns is likely to change bioactive sites and catalytic activities (147). Further, the released *N-*glycans from glycoproteins can be recovered from protein production streams and used as an added source of highly specific prebiotics for the infant gut microbiome. These *N*-glycans are then converted into metabolites with energy value for the infant (e.g., acetate and lactate) when competent *Bifidobacterium* are present, as well as to enhance the colonization of specialized bifidobacteria, such as *B. infantis*, which provide essential ecosystem services to the infant gut (Figure 3) (94).

The ENGase enzymes may be either used in the production step to release high *N*-glycan concentrations in the infant formulations or included as components of designed formulas to release *N*-glycans *in situ* in the gut. Theoretically both native ENGases produced by baby and infant safe organisms such as EndoBI enzymes of *B. infantis* and ENGases produced by recombinant organisms could be used for these applications. However, in practice regulations in most of the globe and especially in EU do not allow the use of GMO in baby and infant products.

Although baby food and infant formula prepared with ENGases produced by recombinant organisms used in the production step could be considered as products derived with GMOs, public and private standards for baby and infant products are too strict to use these products yet for both US and EU markets. Therefore, in the immediate future instead of ENGases produced by recombinant organisms, ENGases produced by baby and infant safe organisms such as EndoBI enzymes of *B. infantis* are more realistic. Although the regulators in the European Union can change the complete regulatory system from a process-based system to a strictly product-based system, such as in Canada in the future, these changes are unlikely to affect baby and infant products.

## Conclusion

The use of ENGase enzymes in the production of infant formula has great potential to increase the nutritional values of formula by releasing additional carbohydrates as sources of energy and substrates from *N-*glycans, a so far underexploited and underappreciated source. Due to their structural similarity to the HMOs, the release of *N-*glycans is likely to be a more successful approach to increase the potential for infant formula to promote colonization of the infant gut by infant-adapted *Bifidobacterium*, leveraging ingredients already present in these formulations and a growing understanding of the microbial enzymes active in the infant gut ecosystem. Finally, deglycosylation of proteins also has the potential to create value-added formulations as well as to have implications on a manufacturing scale.

## Author Contributions

SK organized the general content of the paper. HD was responsible for general editing and organizing the authors as well as the contribution for two sections. MK, AS, and HK contributed one section of the paper. AA and SF were responsible for one section of the paper. All authors contributed to the article and approved the submitted version.

## Conflict of Interest

The authors declare that the research was conducted in the absence of any commercial or financial relationships that could be construed as a potential conflict of interest.
